# Differentially expressed microRNAs in pre-transplant lung biopsies target immune checkpoint proteins and can predict primary graft dysfunction in lung transplantation

**DOI:** 10.1016/j.heliyon.2025.e42515

**Published:** 2025-02-08

**Authors:** Vitale Miceli, Pia Ferrigno, Claudio Centi, Claudia Carcione, Gioacchin Iannolo, Valentina Agnese, Giovanna Lo Iacono, Rosa Liotta, Pier Giulio Conaldi, Massimo Pinzani, Lavinia De Monte, Alessandro Bertani

**Affiliations:** a Research Department, IRCCS ISMETT (Istituto Mediterraneo per i Trapianti e Terapie ad alta specializzazione), Palermo, Italy; b Division of Thoracic Surgery and Lung Transplantation, Chest Center, IRCCS ISMETT (Istituto Mediterraneo per i Trapianti e Terapie ad alta Specializzazione), Palermo, Italy; c Ri.MED Foundation, Palermo, Italy; d Pathology Unit, IRCCS ISMETT (Istituto Mediterraneo per i Trapianti e Terapie ad alta specializzazione), Palermo, Italy; e UPMCI (University of Pittsburgh Medical Center Italy), Palermo, Italy

**Keywords:** Lung transplantation, Primary graft dysfunction, microRNA, Immune checkpoints, Ischemia-reperfusion injury, Prognostic biomarkers, Pre-transplantation biopsy

## Abstract

Lung transplantation (LTx) significantly improves outcomes for patients with end-stage respiratory failure. However, primary graft dysfunction (PGD) remains one of the most relevant hurdles. Although PGD is attributed to ischemia-reperfusion injury (IRI), immune responses, primarily T cell-mediated, may play a pivotal role in its pathogenesis. Additionally, innate immune activation following IRI links PGD to adaptive alloimmunity, highlighting the impact of early events on LTx outcomes. Immune checkpoints (ICPs) such as PD-1/PD-L1, CD40/CD40LG, and OX40/OX40L, regulate post-LTx T cell responses, and dysregulation of microRNAs (miRNAs) has been implicated in altering ICP expression, influencing the amplification of immune responses.

In this preliminary study, we used the taqMan low-density array (TLDA) cards to investigate miRNA dysregulation's prognostic potential as a PGD marker in pre-transplant back-table lung biopsies. Our analysis revealed differential miRNA expression in donor lung tissues, potentially associated with PGD onset, targeting immune regulatory pathways. Specifically, deregulated miRNAs targeted key ICP proteins, including PD-L1, CD40LG, and OX40L. Moreover, the differential expression of these miRNAs was observed in grafts with future PGD compared to grafts without PGD, suggesting a potential prognostic benefit and a possible role for lung tissue miRNAs in the onset of early graft dysfunction.

These findings provide a basis for future investigations into their mechanistic roles and therapeutic potential for PGD. Although based on a limited number of cases, our results imply that miRNAs might be involved in early graft dysfunction. While requiring validation in larger cohorts, our data raise the possibility that the evaluation of the aforementioned markers during the pre-transplant phase, might offer a prognostic benefit in monitoring the onset of PGD. Additionally, the use of compounds that can modulate the function of these molecules could be evaluated for the management of LTx patients.

## Highlights

• Immune checkpoints may be involved in the onset of lung graft dysfunction.
• Dysregulation of miRNAs may impact the expression of immune checkpoints.
• Evaluation of lung miRNAs before transplantation could offer prognostic benefits.

## Graphical abstract

**Figure:**
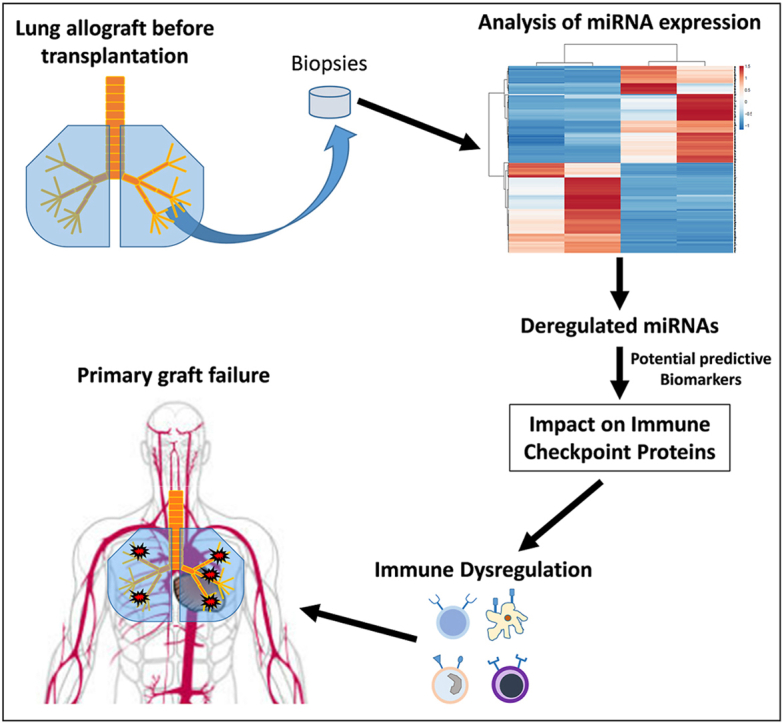

## 1 Introduction

Lung transplantation (LTx) has evolved into a successful therapy for patients with end-stage respiratory failure thanks to advancements in lung preservation techniques, surgical procedures, and immunosuppression [[1], [2], [3]]. One-year survival rates have significantly improved, reaching almost 90 % in current practice, way more favorable than the early stages of lung transplantation in the 1990s [4,5]. However, primary graft dysfunction (PGD), an acute lung injury that occurs early after LTx (24–72 h after surgery) [6], is one of the most common LTx complications related to lung ischemia-reperfusion injury (IRI) [7]. This condition can lead to endothelial activation, monocyte/neutrophil recruitment, and tissue damage, which are strongly associated with mortality [6,[8], [9], [10], [11]]. The exact mechanisms by which PGD occurs and can impact the results of LTx have yet to be elucidated. While certain clinical factors in donors and recipients correlate with PGD, the reasons underlying variable lung damage in distinct patients are unclear [12]. Interestingly, lung allografts that develop PGD have been associated with a hyperinflammatory phenotype [13], which, in the early phase after transplantation, can lead to type 1 T helper (Th1) activation [14]. Recipients with PGD are also more likely to produce anti-HLA antibodies after transplantation, and this can affect their survival [15,16]. Thus, predicting the prognosis after LTx remains a difficult challenge, and the research into the prevention of PGD is highly relevant for improving LTx outcome. The possibility to predict PGD and the related morbidity and mortality [17,18] in transplant patients is still very limited. Prediction of PGD may offer a window of opportunity for timely therapeutic interventions, such as extracorporeal membrane oxygenation (ECMO), before the decline in lung function is clinically detected. Research in this field should be focused on finding minimal or non-invasive predictive biomarkers.

The intricate interplay of immunological factors in the pathogenesis of lung immune dysfunction is still an enigma, despite insights provided by studies on both innate and adaptive immunity [19,20]. The mechanistic understanding of lung allograft dysfunction has been gleaned from small-animal models, shedding light on distinct pathways from other organs [19,21]. Recently, the idea of a tailored immunosuppressive regimen for lung transplantation has been explored, driven by recent discoveries related to immune checkpoint molecules [22,23]. In particular, while some studies investigated the potential beneficial effects of immune checkpoint stimulation treatments to prevent solid organ dysfunction [24,25], immune checkpoint inhibitors have showed clinical benefit for the treatment of malignancies in solid organ transplant recipients but have also displayed an increased risk of transplant dysfunction [26]. Therefore, it becomes crucial to understand the role of immune checkpoint molecules in lung allograft tolerance and dysfunction, where unique immunological features are present.

Scientific evidence has well documented the role of microRNAs (miRNAs) in various lung pathologic processes, and their crucial importance in the progression and maintenance of lung diseases [27], including lung IRI, and dysfunction after LTx [28,29]. MiRNAs are small non-coding RNAs that are highly conserved among species, and modulate the expression of several proteins that control various pathways [30]. Assessing these molecules, not only helps in identifying novel functional miRNAs, but also provides insights into miRNA–protein interactions and their influence on pathways related to pathological processes [31,32]. MiRNA quantification in several biofluids has emerged as a promising new approach for disease biomarker detection and monitoring in the lungs [33,34]. Moreover, the identification of specific signatures and their differential expression may help in distinguishing various outcomes, such as early graft dysfunction associated with rejection and others without rejection [35,36]. Notably, because miRNAs are relatively stable, they are well preserved in a range of sample types, including formalin-fixed tissues [37,38]. This aspect paves the way for new diagnostic approaches.

In this preliminary study, we investigated the expression of miRNAs as potential predictive biomarkers in pre-transplant back-table lung biopsies to enhance the early diagnosis and prognosis of PGD. Molecular analyses aim to characterize miRNA profiles that might affect, at least in part, the onset of PGD.

## 2 Methods

### 2.1 Study population and sample collection

We conducted an observational, retrospective pilot study on archived formalin-fixed paraffin-embedded lung tissue blocks obtained during the back-table phase of consecutive lung transplants performed at IRCCS ISMETT from June 21, 2007 to July 10, 2012. This study was approved by IRCCS ISMETT's institutional review board (ref. IRRB/46/21), and written informed consent was obtained. Clinical outcome data were recorded in the IRCCS ISMETT lung transplant database. To analyze a homogeneous cohort of patients without minimal bias that could affect our findings, we selected a cohort of crossmatch-negative patients who survived the subsequent 10 years of follow-up. Moreover, all patient sera were tested for the presence of donor‐specific antibodies (DSAs) as described previously [39] and were all negative. PGD scores were retrospectively assigned based on the 2016 International Society for Heart and Lung Transplant (ISHLT) guidelines. PGD scores were determined at different time points, including T0, T24, T48, and T72 h, which correspond to 6, 24, 48, and 72 h after lung reperfusion. In order to distinguish patient with or without PGD, we considered the outcome variable as PGD 0 *versus* any PGD value at any time.

### 2.2 miRNA expression profiling

We analyzed miRNA expression in 20 μm thick sections of each sample with TaqMan® low-density arrays (TLDA) (TaqMan® Array Human MicroRNA A + B Cards Set v3.0) according to the manufacturer's instructions (Thermo Fisher Scientific, Waltham, U.S.A.). Three FFPE tissue sections in micro-centrifuge tubes were deparaffinized in a deparaffinization solution (Qiagen, Hilden, Germany), and total RNA was extracted with miRNeasy FFPE Purification Kit according to the manufacturer's instructions (Qiagen, Hilden, Germany). Both purity and quantity were determined by OD260/280 using a NanoDrop Spectrophotometer (Thermo Fisher Scientific, Waltham, U.S.A.). RNA was reverse-transcribed and pre-amplified with Megaplex™ Pools Protocol according to the manufacturer's instructions (Thermo Fisher Scientific, Waltham, U.S.A.). We analyzed the expression of 754 human miRNAs with the QuantStudio™ 7 Pro Real-Time PCR System (Thermo Fisher Scientific, Waltham, U.S.A.). The fold change in miRNA expression was determined with the 2^DDCt method, using the U6 as reference gene. Hierarchical cluster analysis of miRNA expression was used to group patients. In particular, miRNA expression data were grouped using the Cluster 3.0 program, and a heat map was generated using the Java TreeView program. Differentially expressed miRNAs (DEMs) were hierarchically clustered using an average linkage algorithm, and a Euclidean distance and the R functions (v4.1.2) were used to do principal component analysis (PCA).

### 2.3 miRNA target prediction and functional analysis

First, we performed miRNA enrichment analysis in the Reactome terms of the 30 significantly differentially expressed miRNAs (obtained after volcano plot analysis with p < 0.05 and fold change >1.5) with the online database miRNA Enrichment Analysis and Annotation Tool 2.0 (miEAA 2.0) [40] (https://ccb-compute2.cs.uni-saarland.de/mieaa/, accessed on January 15, 2024). MiEAA integrates data from different sources, including Gene Ontology, miRBase, Reactome, and miRTarBase, as well as other datasets, and is based on GeneTrail [41]. We then used the miRNet online database [42] (https://www.mirnet.ca/, date of access: January 15, 2024) to build predicted interactions between miRNAs and target genes. The miRNet database is a comprehensive atlas of miRNA–target interactions that can integrate the information resulting from 11 existing miRNA–target prediction programs (TarBase, miRTarBase, miRecords, miRanda, miR2Disease, HMDD, PhenomiR, SM2miR, PharmacomiR, EpimiR, and starBase). Each software allowed for the elucidation of molecular pathways controlled by miRNAs. Specifically, with miRNET we investigated the functional implications of miRNA deregulation in the function of ICPs related to the adaptive immune system, and generated a protein–protein interaction network of proteins targeted by deregulated miRNAs, and potentially involved in crucial pathways of PGD.

### 2.4 Statistical analysis

Anthropometric and clinical characteristics of patients are reported as mean ± SD for continuous data and frequencies, and percentages for categorical data. Statistical differences between patients with PGD and without PGD were analyzed with parametric Student's t-test. To examine the significant differences of both miRNA and protein expression between samples, we did either parametric Student's t-test or a non-parametric Mann–Whitney *U* test. An appropriate paired or unpaired Student's t-test was used for the comparison of fold change values (2^DDCt). We also analyzed multiple pair-wise comparisons of each 2^DCt value per group through one-way ANOVA (GraphPad Prism 6.0, San Diego, U.S.A.). P of <0.05 was considered significant. We clustered and correlated data using hierarchical clustering and Euclidean distance method. Moreover, both sensitivity and specificity of miRNAs to discriminate between healthy and PGD patients were assessed with receiver operating characteristic (ROC) curve analysis.

## 3 Results

### 3.1 Recipient characteristics

For this exploratory study, a total of 8 patients who underwent bilateral lung transplantation and 2 patients who underwent single lung transplantation were selected for analyses. Clinical and demographics characteristics of donors and recipients (n = 10) are summarized in Table 1. Mean donor age was 39.1 years (±11.15), and mean age for recipients was 47.47 (±16.19). The recipients were 8 men and 2 women, 4 of whom suffered from restrictive lung disease, 3 from COPD, 2 from cystic fibrosis, and 1 from bronchiectasis. Among patients who developed PGD and those who did not, statistical significance was not reached for several variables, such as age of the recipient and donor, crossmatch, immunosuppression (except for mycophenolate mofetil treatment), and presence of DSAs, likely due to sample size. Among the 10 patients included in the study, 2 (20 %) had grade 1 PGD, 2 (20 %) had grade 2 PGD, and 1 (10 %) had grade 3 PGD, which developed between 48 and 72 h. Of the 10 cases analyzed (both with PGD and without PGD) none died after 1, 5 or 10 years.

**Table 1:** Demographics and clinical characteristics of donors and lung transplant recipients comprising the study cohort.

Variable	TOTAL	NO PGD AT T48-72 h	PGD AT T48-72 h	P value
**Donor characteristics**				
Age (years)	39.1 ± 11.15	35.8 ± 13.80	42.4 ± 7.89	0.38
Women	7 (70 %)	3 (60 %)	4 (80 %)	0.54
Men	3 (30 %)	2 (40 %)	1 (20 %)	0.54
Intubation (hours)	60 ± 20.39	57.6 ± 21.47	62.4 ± 21.46	0.73
pO2 (fiO2 100 %)	510.2 ± 51.57	527.6 ±	492.8 ± 68.88	0.31
Smoker >20PYH	1 (10 %)	1 (20 %)	0 (0 %)	0.35
**Recipient characteristics**				
Age (years)	47.47 ± 16.19	48.748 ± 24.01	46.19 ± 3.12	0.82
Women	2 (20 %)	1 (20 %)	1 (20 %)	1.00
Men	8 (80 %)	4 (80 %)	4 (80 %)	1.00
Grade 1 PGD	2 (20 %)	0 (0 %)	2 (40 %)	0.14
Grade 2 PGD	2 (20 %)	0 (0 %)	2 (40 %)	0.14
Grade 3 PGD	1 (10 %)	0 (0 %)	1 (20 %)	0.35
**Primary disease**				
Restrictive lung disease	4 (40 %)	2 (40 %)	2 (40 %)	1.00
Cystic fibrosis	2 (20 %)	1 (20 %)	1 (20 %)	1.00
Bronchiectasie	1 (10 %)	0 (0 %)	1 (20 %)	0.35
COPD	3 (30 %)	2 (40 %)	1 (20 %)	0.54
LAS	34.07 ± 3.69	34.424 ± 4.70	33.49 ± 1.66	0.76
**Condition at time of transplant**				
Mean pulmonary artery pressure (mPAP)	36.9 ± 30.62	42.2 ± 41.57	31.6 ± 17.67	0.61
mPAP >20 mmHg	9 (90 %)	4 (80 %)	5 (100 %)	0.35
DSAs (subjects, n)	0 (0 %)	0 (0 %)	0 (0 %)	1.00
Crossmatch (subjects, n)	0 (0 %)	0 (0 %)	0 (0 %)	1.00
**Immunosuppression**				
Pred/Tacro/Basi	10 (100 %)	5 (100 %)	5 (100 %)	1.00
+MMF	3 (30 %)	0 (0 %)	3 (60 %)	0.04
**Operative characteristics**				
Type of transplant bilateral	8 (80 %)	5 (100 %)	3 (60 %)	0.14
Intraoperative support ECLS	6 (60 %)	3 (60 %)	3 (60 %)	1.00
Total ischemic time (min)	367.1 ± 72.25	390.2 ± 68.61	344 ± 75.53	0.34
**Outcomes**				
Post-op length of stay (days)	35.2 ± 17.44	41 ± 22.66	29.4 ± 9.34	0.32
Mechanical ventilation ≥5 days	1 (10 %)	1 (20 %)	0 (0 %)	0.35
Reintubated	4 (40 %)	1 (20 %)	3 (60 %)	0.24
CLAD	2 (20 %)	0 (0 %)	2 (40 %)	0.14
1-year mortality	0 (0 %)	0 (0 %)	0 (0 %)	1.00
5-year mortality	0 (0 %)	0 (0 %)	0 (0 %)	1.00
10-year mortality	0 (0 %)	0 (0 %)	0 (0 %)	1.00

Values are n (%) or mean ± SD. PGD, primary graft dysfunction; CLAD, chronic lung allograft dysfunction; COPD, chronic obstructive pulmonary disease; LAS, lung allocation score; ECLS, extracorporeal life support; DSAs, donor‐specific antibodies; Pred, prednisolone; Tacro, tacrolimus; Basi, basiliximab; MMF, mycophenolate mofetil.

### 3.2 Analysis of differentially expressed miRNAs in PGD compared to non-PGD patients

**Fig. 1:**
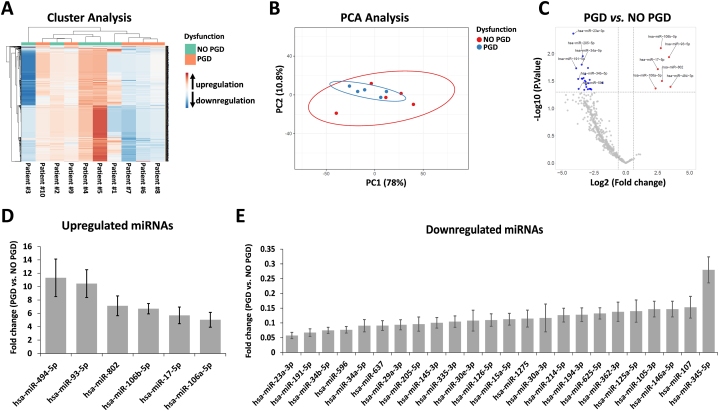
RT-PCR analysis of miRNAs in lung grafts with or without future primary graft dysfunction (PGD). (A) Hierarchical clustering based on miRNA expression levels of 768 miRNAs. Heatmap colors represent relative miRNA expression normalized to housekeeping. (B) PCA analysis of miRNA expression levels. (C) Volcano plot analysis of deregulated miRNAs obtained between PGD and no PGD patients (p < 0.05 and fold change >1.5). (D) Upregulated miRNAs in PGD vs. no PGD patients. (E) Downregulated miRNAs in PGD vs. no PGD patients. MiRNA levels were normalized to those of U6. Data are means ± SD.

To identify DEMs associated with lung graft dysfunction, we profiled the expression of 768 miRNAs isolated from pre-LTx back-table graft biopsies independently from their PGD status. A preliminary evaluation of miRNA expression was made by hierarchical clustering analysis. This showed dissimilar expression patterns between lungs that developed PGD and those that did not, as well as between patients in each group (PGD or no PGD group) (Fig. 1A). We investigated the same data through principal component analysis (PCA) and found that no significant variations were observed between PGD and no PGD patients (Fig. 1B). We then performed a volcano plot analysis (fold change >1.5 and p < 0.05) (Fig. 1C) and found that 6 miRNAs were significantly upregulated (Fig. 1D), and 24 miRNAs were significantly downregulated (Fig. 1E) in biopsies of lungs that developed PGD compared to non-PGD biopsies.

### 3.3 Target gene prediction

**Fig. 2:**
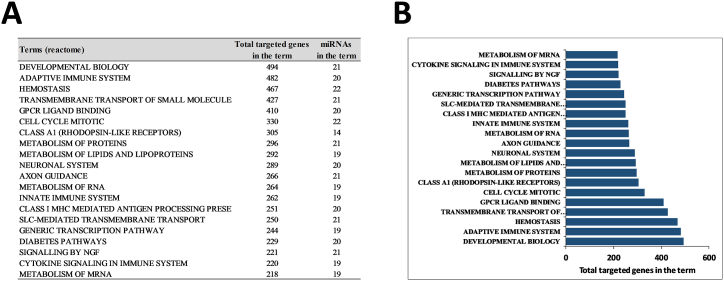
Partial list of GO enrichment analysis (GO category analyzed by MiEAA tool) for the 30 deregulated miRNAs in PGD vs. no PGD patients categorized by total targeted genes.

**Fig. 3:**
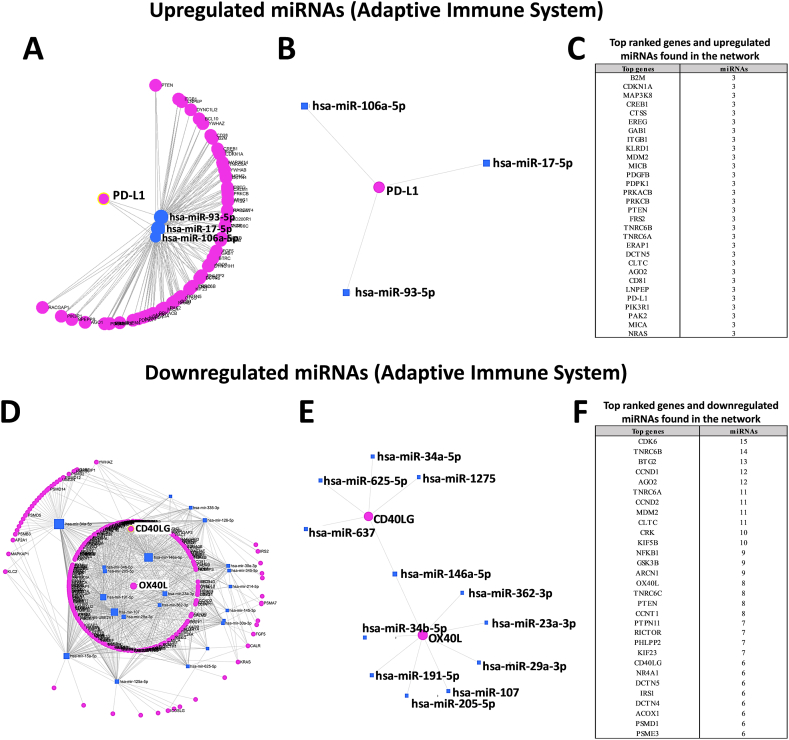
Protein-protein interaction network generated for 30 significantly deregulated miRNAs and target genes after miRNet analysis. Only genes targeted by at least three significant differentially expressed miRNAs (DEMs) are shown. (A, B and C). Images show networks with all interactions between significantly upregulated miRNAs and genes involved in adaptive immune system. (D, E and F) Images show networks with all interactions between significantly downregulated miRNAs and genes involved in adaptive immune system.

In order to study the role of significantly DEMs in the graft dysfunction, we did a gene ontology (GO) enrichment analysis with the online miEAA tool. Among the top-ranked terms we found, we observed that 20 of the 30 DEMs targeted 482 genes belonging to the term “adaptive immune system” (the second top-ranked term shown in Fig. 2A and B). Thus, to further study the involvement of DEMs in the regulation of immune processes enabling protective immunity or tolerance, we analyzed DEMs for the term “adaptive immune system” using the miRNet online tool. Among the top-ranked genes targeted by upregulated miRNAs, we observed that 3 of 6 miRNAs targeted the crucial immunosuppressive checkpoint protein PD-L1 (Fig. 3A–C). Moreover, in the top ranked genes targeted by downregulated miRNAs, 12 of 24 miRNAs targeted 2 key stimulatory immune checkpoint proteins, CD40L and OX40L (Fig. 3D–F).

**Fig. 4:**
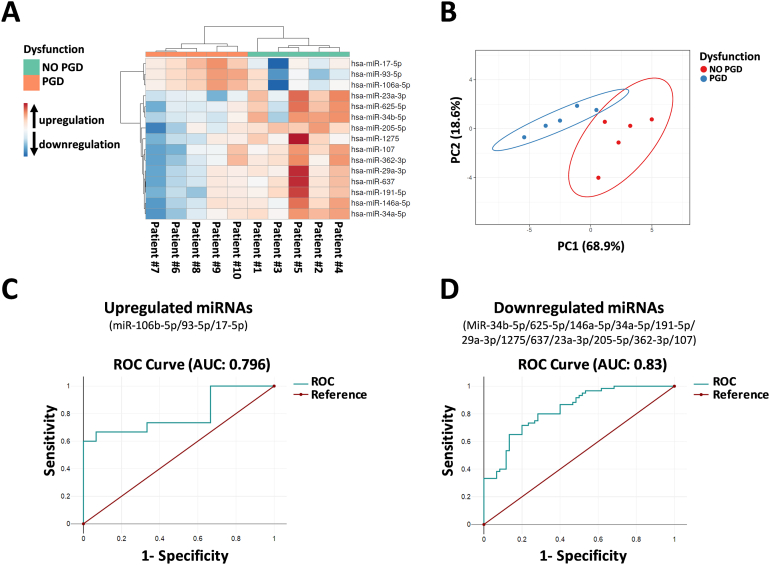
RT-PCR analysis of differentially expressed miRNAs (DEMs) in lung grafts with or without future primary graft dysfunction (PGD). (A) Hierarchical clustering based on the expression levels of 15 significant DEMs. Heatmap colors represent relative miRNA expression normalized to housekeeping. (B) PCA analysis of miRNA expression levels. (C) ROC curves of the prognostic potential of significantly upregulated miRNAs (miR-106b-5p, miR-93-5p, miR-17-5p), and (D) downregulated miRNAs (miR-34b-5p, miR-625-5p, miR-146a-5p, miR-34a-5p, miR-191-5p, miR-29a-3p, miR-1275, miR-637, miR-23a-3p, miR-205-5p miR-362-3p, miR-107). MiRNA levels were normalized to those of U6. Data are means ± SD.

We performed hierarchical cluster analysis of the 15 DEMs targeting the aforementioned ICP proteins, revealing that these miRNAs were capable of grouping patients in relation to PGD (Fig. 4A). These data were confirmed by PCA analysis (Fig. 4B). We also evaluated the prognostic power of both upregulated and downregulated miRNAs through ROC analyses for prediction of PGD occurrence, and a scoring approach revealed a good prognostic accuracy, with an AUC ranging between from 0.796 to 0.83 (Fig. 4C and D).

## 4 Discussion

Following solid organ transplantation, the activation of innate immunity occurs primarily in response to IRI, during which the production of several chemokines and cytokines leads to the recruitment of immune cells into the allograft [43]. Lungs are particularly susceptible to IRI, a condition that can lead to PGD [44], which is often associated with an increased likelihood of mortality [6,8,45,46]. IRI typically triggers inflammatory processes mediated by neutrophils. These cells amplify graft injury by releasing pro-inflammatory mediators, which stimulate T lymphocyte activation and inhibit tolerance induction [[47], [48], [49], [50]]. This cascade establishes a critical link between PGD and adaptive alloimmune responses. In fact, the clinical phenotype with increasing and late PGD (grade 3), was defined as the immunological phenotype, and reveals the activation of innate and adaptive immunity, leading to progressive endothelial-epithelial alveolar injury during the early phase after transplantation [51]. During this process, neutrophils and lymphocytes contribute to lung graft damage, but also play roles in tissue repair. For instance, neutrophils can facilitate repair through efferocytosis [52], while T lymphocytes can contribute to inflammation through antigen-independent cytokine and chemokine production [53]. Moreover, early innate immune signals can trigger the lymphocytes to carry out the later adaptive immune response [51]. The balance between these processes probably defines the severity of lung IRI and the PGD resolution [51], and the long-term survival of transplanted lungs may be a function of the early immune response. This suggests that early immunological events within the lung allograft can lead to lung dysfunction and set the stage for allorecognition [47,48,54,55].

The PD-1/PD-L1 signaling pathway serves as a crucial regulatory mechanism able to inhibit reactive T cells, influencing their role in the amplification of immune responses after LTx [56]. Conversely, a different immune checkpoint pathway, such as CD40/CD40LG, represents a crucial mechanism involved in the activation of reactive T cells. Indeed, the soluble form of CD40LG or CD40LG cell-surface protein, binding to CD40 of T cells, results in promoting antigen presentation and cytokine production for further activation of both T and B cells [57,58]. These data were confirmed in the cardiac allograft rat model study by Hancock et al., who demonstrated that by administrating an anti-CD40LG in donors through transfusions, allograft function was extended beyond 150 days [59]. In a mouse model, it has also been shown that the interaction of ICPs such as OX40L and OX40 is a crucial pathway for inhibition of the tolerance mainly mediated by human T cells. OX40L can provide a synergistic costimulatory signal for antigen-reacting naive CD4 T cells, inducing an increase of inflammatory cytokines [60]. In this regard, *in vivo* studies have found that treatment with an agonist antibody for OX40 enhances the generation of antigen-specific effector T cells, preventing the induction of T cell tolerance [[60], [61], [62]].

Interestingly, it was revealed that the regulation of many T cell functions can be affected by miRNAs [63], and the development of lung dysfunction following LTx can induce differential expression of miRNAs, influencing overall immunity [29]. These important findings highlight the potential regulatory abilities of miRNAs in the intricate mechanisms that can lead to post-transplant organ dysfunction. Moreover, these processes can also occur in the early stages of transplantation and might be involved in the development of PGD.

In this preliminary study, we hypothesized that abnormal miRNA expression in lung tissues before transplantation, also due to IRI, might govern some mechanisms leading to PGD after transplantation. To explore this hypothesis, we analyzed the expression of miRNAs in lung back-table, pre-implantation biopsies, and investigated their role using gene ontology analysis. We found that 30 significant DEMs in grafts with future PGD (Fig. 1D–E) target genes involved in immune system regulation processes, including “adaptive immune system,” “innate immune system,” “class I MHC mediated antigen processing presentation,” and “cytokine signaling in immune system” (Fig. 2). Among these processes, adaptive immune system pathways (the second top-ranked term) might be involved, at least in part, in the onset of PGD [39,44,64,65]. In this regard, our results provide evidence that 15 DEMs might induce a dysregulation of specific ICPs, including PD-L1, CD40LG, and OX40L (Fig. 3). Moreover, our study also showed that both cluster analysis and PCA of the 15 aforementioned miRNAs were able to group the patients of our cohort in relation to the presence or absence of PGD (Fig. 4A–B), and ROC analyses revealed good prognostic accuracy for both upregulated (AUC 0.796) and downregulated miRNAs (AUC 0.83) (Fig. 4C–D). Notably, PD-L1, which plays a crucial role in suppressing T cell-mediated immune responses [66], was targeted by 3 miRNAs (miR-106a-5p, miR-17-5p, miR-93-5p) that we found downregulated in non-PGD patients compared to patients with lung dysfunction (Fig. 3B). Conversely, 12 miRNAs (miR-34a-5p, miR-625-5p, miR-1275, miR-637, miR-146a-5p, miR-362-3p, miR-23a-3p, miR-29a-3p, miR-107, miR-205-5p, miR-191-5p, miR-34b-5p) were downregulated in PGD patients, and targeted both CD40LG and OX40L (two important co-stimulatory ICPs) (Fig. 3E). PD-L1, CD40LG, and OX40L-mediated pathways are crucial in controlling the level of T cell responses, leading, in the case of transplantation, to the amplification of immune responses [[56], [57], [58], [59], [60], [61], [62],^67^].

Based on the data from this study, we can hypothesize that back-table lung biopsies’ analysis revealed differential miRNA expression potentially associated with PGD, targeting proteins involved in immune regulation processes, including PD-L1, CD40LG, and OX40L, potentially activating T cells and possibly contributing to the onset of PGD. Abnormal miRNA expression in lung tissues before transplantation, also due to IRI, might govern immune mechanisms crucial to determine the onset of PGD, such as immune checkpoint signaling, which can drive graft inflammation after transplantation. Indeed, the stimulation of T cells, has been suggested as a crucial mechanism in the onset of lung allograft acceptance. In particular, using a murine model of LTx, Krupnick et al. found that the early infiltration of CD8 central memory T cells was crucial for lung allograft tolerance [68]. In the same animal model, it was demonstrated that, after transplantation, alloreactive T cells are trigged directly within the lung allografts and not in secondary lymphoid organs [69], suggesting a direct interaction and regulation between graft and T cells. Recently, it has been confirmed that central memory CD8 T lymphocytes are crucial in tolerance induction after lung transplantation, and the PD-1/PD-L1 signaling in reactive CD8 T cells is a crucial pathway that influences the role of CD8 cells in tolerance induction after LTx [56]. Moreover, the binding of PD-L1 to PD-1 on helper T cells has been shown to stimulate effector T cell differentiation into regulatory T cells, with consequent decreases in inflammatory cytokine production [70].

Early pathophysiologic events within the lung allograft, including miRNA dysregulation, might influence lung parenchymal phenotype and early immune cell processes crucial in graft dysfunction. If miRNAs are confirmed by further studies as predictive biomarkers of PGD, they could be used to improve the management of PGD patients. We acknowledge that PGD could be occurring as a result of both donor and recipient determinants. Furthermore, the main limitation of our preliminary study is the small sample size, which may limit the generalizability of the results. Nevertheless, the clinical features of our cohort of patients were acceptably balanced and patients did not experience pre- or post-operative obvious surgical complications. Our panel of predictive biomarkers was far from exhaustive with regard to exploring all the relevant immune processes involved in lung dysfunction but, mechanistically, the interplay between miRNA expression and immunosuppressive/stimulatory ICPs suggested by our results, adds new insight into the biomedical research of LTx. Although ICPs are currently known as key contributors to allorecognition and implicated in chronic rejection of lung grafts [23,71], early T cell-mediated events driven by ICPs might play a role in the processes underlying PGD immediately following transplantation. Our data, which will need to be validated in larger cohorts of patients, shed light on the interplay between miRNA expression and ICPs in back-table biopsies before transplantation, and this might offer a prognostic advantage in monitoring the onset of early graft dysfunction, improving the management of LTx patients.

## CRediT authorship contribution statement

**Vitale Miceli:** Writing – original draft, Visualization, Validation, Investigation, Formal analysis, Conceptualization. **Pia Ferrigno:** Investigation. **Claudio Centi:** Investigation. **Claudia Carcione:** Investigation. **Gioacchin Iannolo:** Investigation. **Valentina Agnese:** Investigation. **Giovanna Lo Iacono:** Investigation. **Rosa Liotta:** Investigation. **Pier Giulio Conaldi:** Writing – review & editing, Supervision, Project administration. **Massimo Pinzani:** Writing – review & editing. **Lavinia De Monte:** Investigation. **Alessandro Bertani:** Writing – review & editing, Validation, Supervision, Project administration, Investigation, Formal analysis, Conceptualization.

## Ethics statement

The study was conducted according to the ethical guidelines of the Helsinki Declaration and was approved by the IRCCS ISMETT's institutional review board (project identification code: IRRB/46/21). Written informed consent was obtained from individual or guardian participants.

## Data availability statement

The data associated with this study have not been deposited in a publicly accessible repository. All of the relevant data are available from the corresponding author on reasonable request.

## Funding

This research was funded by the 10.13039/501100003196Italian Ministry of Health, Ricerca Corrente.

## Declaration of competing interest

The authors declare that they have no known competing financial interests or personal relationships that could have appeared to influence the work reported in this paper.
